# Cerebral Small Vessel Disease and Alzheimer's Disease: A Review

**DOI:** 10.3389/fneur.2020.00927

**Published:** 2020-08-25

**Authors:** Hae Won Kim, Jeongho Hong, Jae Cheon Jeon

**Affiliations:** ^1^Department of Nuclear Medicine, Keimyung University Dongsan Medical Center, Daegu, South Korea; ^2^Department of Neurology, Keimyung University Dongsan Medical Center, Daegu, South Korea; ^3^Institute for Medical Science, Keimyung University School of Medicine, Daegu, South Korea

**Keywords:** cerebral small vessel disease, Alzheimer's disease, dementia, white matter hyperintensity, PET

## Abstract

Alzheimer's disease (AD) is the most common cause of dementia. Despite this, clear pathophysiology for AD has not been confirmed, and effective treatments are still not available. As AD results in a complex disease process for cognitive decline, various theories have been suggested as the cause of AD. Recently, cerebral small vessel disease (SVD) has been suggested to contribute to the pathogenesis of AD, as well as contributing to vascular dementia. Cerebral SVD refers to a varied group of diseases that affect cerebral small arteries and microvessels. These can be seen as white matter hyperintensities, cerebral microbleeds, and lacunes on magnetic resonance imaging. Data from epidemiological and clinical-pathological studies have found evidence of the relationship between cerebral SVD and AD. This review aims to discuss the complex relationship between cerebral SVD and AD. Recent reports that evaluate the association between these diseases will be reviewed.

## Introduction

Alzheimer's disease (AD) is the most common cause of dementia, accounting for about 60% of all dementia cases (1). As AD results in a complex disease process for cognitive decline, various theories have been suggested as the cause of AD in many epidemiological, biochemical, genetic, and animal studies. The main hypothesis, to date, is the amyloid-β (Aβ) cascade hypothesis, which is that Aβ is the most important factor in the pathogenesis of AD (2). Along with the Aβ cascade hypothesis, another major theory is the tau hypothesis, in which the abnormal phosphorylation of tau protein results in paired helical filament tau and neurofibrillary tangles, causing neurodegeneration (3). However, the clear pathophysiology for AD, detailing the contributions of cerebral Aβ accumulation and abnormal phosphorylation of tau protein has not been confirmed, and effective treatments are still not available (4).

Cerebral small vessel disease (SVD) refers to a varied group of diseases that affect the cerebral small arteries and microvessels. These can be seen as white matter hyperintensities (WMHs), cerebral microbleeds (CMBs), and lacunes on magnetic resonance imaging (MRI) (5). Cerebral SVD is the most common pathological neurological process and has an important role in dementia as well as strokes (5). Since the causes of AD were first explored, studies have focused on the relationship between AD and cerebral SVD (6, 7). Recently, it has been hypothesized that cerebral SVD contributes to the pathogenesis of both AD and vascular dementia (8, 9). AD has similar risk factors to cerebral SVD, such as hypertension and diabetes (10) as well as pathophysiological mechanisms such as oxidative stress, inflammation, mitochondrial disruption, and metabolic dysfunction (11). For these reasons, the clinical differentiation of AD from vascular cognitive impairment or vascular dementia can be unclear.

Data from epidemiological and clinical-pathological studies have supported a relationship between cerebral SVD and AD, although the role of cerebral SVD in causing AD is still unclear. This review aims to discuss the complex relationship between AD and cerebral SVD. Recent reports that evaluate the association between these diseases will be reviewed. The direction of future research will be also presented by exploring the underlying mechanism of cerebral SVD on AD development and hypotheses will be suggested.

## Review of the Literature

**Figure 1:**
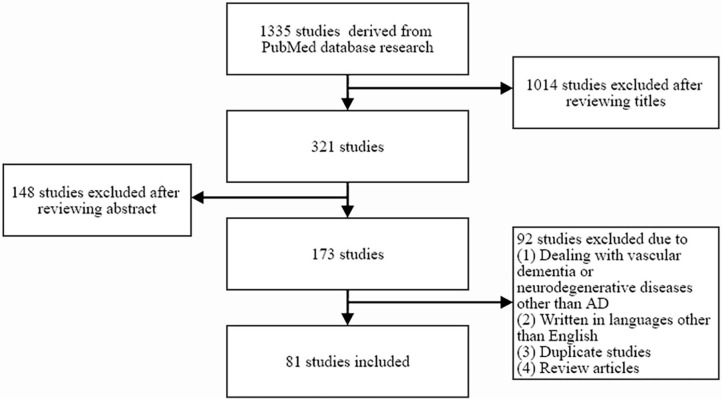
Flow diagram showing the inclusion and exclusion of relevant studies.

By searching the PubMed database (1982–2020), 1,335 potentially relevant studies were identified. The following combinations of keywords were searched: “cerebral small vessel disease” or “white matter hyperintensities” or “microbleed” or “lacunes” and “AD.” A study was selected from the initial search if it described at least one case of cerebral SVD and evaluated the relationships between cerebral SVD and AD. Studies on the relationship between cerebral SVD and broad spectrum of dementia, or studies mainly dealing with vascular dementia or neurodegenerative diseases other than AD, studies written in languages other than English, duplicate studies, and review articles were excluded. A total of 81 studies were selected for inclusion by reviewing the titles and abstracts of identified articles (Figure 1).

## Cerebral Small Vessel Disease

Cerebral SVD refers to a varied group of diseases that involve the small (40~250 μm) perforating arterioles, capillaries, and venules of the brain, causing various lesions that can be seen on pathological examination or brain imaging (12). Cerebral SVD is typically seen as WMHs, CMBs, lacunes, dilated perivascular spaces, and microinfarcts on MRI (5). WMHs are usually seen as bilateral, mostly symmetrical hyperintensities on T2 MRI in older individuals (5). Pathological studies have shown that WMHs are accompanied by vessel wall thickening, enlargement of perivascular spaces, a decrease in vascular density, and an increase in vessel tortuosity. WMHs are further characterized by demyelination, gliosis, fiber loss, and decreased number of oligodendrocytes (13, 14). The mechanisms for developing WMHs are presumed to be chronic ischemia, blood-brain barrier (BBB) breakdown, dysfunction of oligodendrocyte precursor cells, and venous collagenosis (13, 15, 16). CMBs are usually seen as small areas (<10 mm in diameter) of the signal void with associated blooming on the T2 MRI (5). CMBs have also been known to be associated with various degrees of gliosis and tissue loss (13). Histopathological studies have shown that most CMBs have parenchymal micro-hemorrhages with vessel wall disruption, but some only have vasculopathy, such as vessel wall dissection, microaneurysms, and vessel wall thickening, without hemorrhage (17). A lacune is a round, ~3–15 mm sized, fluid-filled cavity caused by an acute cerebral infarction or a cerebral hemorrhage in subcortical regions within the territory of a perforating arteriole (5). It is usually seen as a central cerebrospinal fluid (CSF)-like hypointensity with a surrounding rim of hyperintensity on a fluid-attenuated inversion recovery (FLAIR) MRI (5). Histopathological studies have shown that the lacune is an irregularly shaped cystic cavity with surrounding mild axonal loss and minimal gliosis (13).

Historically, cerebral SVD has been shown to be related to age-related changes and was thought to be a slowly progressing disease that affected the frontal–subcortical networks, which lead to corresponding frontal symptoms (18). However, this concept of cerebral SVD has evolved because it became clear the spectrum of cognitive symptoms attributable to cerebral SVD is more diverse than that of the classic concept which includes loss of executive control, and decreased speed of behavioral performance. In addition to the classic concept, it includes deficits in language, memory, attention, and visuospatial abilities (13, 19). Recent studies on brain connectomics and functional neuroanatomy have resulted in a better understanding of the mechanism for cerebral SVD in developing the broad spectrum of cognitive symptoms by disrupting the structural or functional connected cerebral networks (13). The structural network was disrupted due to decreases in the number, strength and efficiency of connections in patients with cerebral SVD (20, 21). It has been reported that the degree of brain network disruption is associated with the severity of cerebral SVD, such as WMH volume, number of CMBs, and the number of lacunes (21). Also, the disruption of the structural network in functional neuroimaging studies has shown the disruption of functional connectivity across distributed networks in patients with cerebral SVD (13, 21). The disruption of functional connectivity by cerebral SVD affects the default mode, dorsal attention and frontoparietal networks, resulting in decreased attention and impaired executive functions (22). Patients with cerebral SVD have a lesser degree and later onset of memory impairment compared with AD patients (13). In the context of the functional connectivity disrupted by cerebral SVD, memory impairment has been explained to be a result of impaired executive function, leading to working memory deficits, in turn affecting memory function (23). However, as the relationship between cerebral SVD and AD pathology has been reported (6, 8), cerebral SVD seems to have a direct and synergistic effect on memory function in AD patients.

## Relationship Between AD Risk and Cerebral SVD

Several studies have evaluated the associations between AD risk and cerebral SVD, including WMHs, CMBs, and lacunes. Although the relationship between WMHs and AD is controversial, most of the studies showed that cerebral SVD had a predictive effect on AD risk in older individuals. The Cardiovascular Health Study Cognition Study, which contained 3,375 participants, reported that a significantly increased risk of AD [HR = 1.5 (1.17–1.99)] was observed in individuals with higher grades of WMH over 8 years (24). Hertze et al. (25) reported that the presence of pathologic tau and WMHs in MCI patients was associated with an increased risk of developing AD dementia. This result suggests that while WMHs independently induce cognitive impairment, such impairments are synergistically exacerbated in the presence of pathologic tau. A 3 years follow-up study with 169 MCI patients reported that patients with higher volumes of WMHs in the parietal lobe had more advanced AD progression than those with lower WMH volumes [HR = 1.07(0.99–1.16)] (26). Also, a study by the Clinical Research Center for Dementia of South Korea using 622 participants with MCI reported that severe periventricular WMHs predicted incident all-cause dementia [HR = 2.22 (1.43–3.43)] and AD [HR 1.86 (1.12–3.07)] (27). A longitudinal study with MCI patients from the Alzheimer's Disease Neuroimaging Initiative (ADNI) showed that patients whose symptoms progressed from MCI to AD dementia exhibited increased WMH burden faster than those with stable cognitive function (28). A recent longitudinal study with 2,626 initially cognitively normal subjects showed that high WMH burden was associated with an increased risk of developing AD in a 5 years follow-up period [HR = 1.75 (1.37–2.01)] (29). Furthermore, a recent cohort study with 575 cognitively unimpaired participants revealed that WMH burden was associated with AD risk factors, including cardiovascular risk, age, hypertension, hypercholesterolemia and body mass index, suggesting that control of modifiable risk factors could have a significant impact on AD development (30). However, other studies could not find any significant relationship between WMHs and the prevalence of AD (31–33).

In evaluating the associations between AD risk and CMBs, most studies have failed to find a significant relationship between AD risk and CMBs. A longitudinal study with 729 Japanese participants with vascular risk factors showed that overall CMBs were significantly related to the risk of all-cause dementia [HR = 2.72(1.45–4.93)], but not significantly related with AD risk (34). A meta-analysis of 3 studies on CMBs and AD risk also revealed no significant effect of CMBs on AD incidence using random-effect models (11). In evaluating the associations between AD risk and lacunes, the results of several studies conflict. The Cardiovascular Health Study with 5,888 adults showed a significant relationship between lacunes and the progression of AD [OR = 2.7 (1.0, 7.1)] (32). Contrastingly, two longitudinal studies (34, 35) and three cross-sectional studies (36–38) did not show any significant correlation between lacunes and AD risk. A meta-analysis of these six studies showed that lacunes significantly increased the risk of AD [OR = 1.203 (1.014–1.428)] (11).

Studies evaluating the associations between cerebral SVD and AD risk based on the clinical AD diagnosis with a cognitive function test usually show contradictory results, because cognitive function can be substantially affected by several factors, such as neurodegenerative disease, genetics, physical activity, education level, alcohol abuse, diabetes and cardiovascular disease (39). To clarify the effects of cerebral SVD on the development of AD, longitudinal studies that evaluate pathological changes, such as tau protein or Aβ plaque deposition, rather than studies based on the diagnosis of clinical dementia, are needed.

## Genetic Contributions to Both AD and Cerebral Small Vessel Disease

Genetics affect AD risk. Familial autosomal-dominant genes (*PSEN1, PSEN2*), and amyloid precursor protein (*APP*) gene have been reported as being associated with early-onset AD (40). In addition, there are several genetic markers that influence both AD and cerebral SVD. It is unknown whether these genes cause development of cerebral SVD and indirectly affect AD, or directly affect both SVD and AD. The ε4 allele of the apolipoprotein E *(APOE)* gene is a risk factor for both AD and cerebral SVD. The APOE protein performs several functions, and is critically involved in the development of a number of metabolic, cardiovascular, and neurodegenerative diseases. APOE enables lipid transport by acting as a major cholesterol carrier; one of its primary functions is to help the binding of lipoproteins or lipid complexes to surface receptors of cells in plasma (41). It has three major isoforms *(APOE* ε*2, APOE* ε*3, and APOE* ε*4)* with different effects on lipid and neuronal homeostasis. In Caucasian populations, individuals with the *APOE* ε*4* allele were reported to have a 10-fold higher risk of developing AD than those without the *APOE* ε*4* allele (42). APOE is involved in the breakdown and tau-mediated neurodegeneration of cerebral Aβ plaques. *APOE* ε*4* does not efficiently perform a breakdown of Aβ plaques, and is less efficient than other alleles in maintaining cerebral homeostasis of lipid transport, synaptic integrity, glucose metabolism, and cerebrovascular function (43). Also, the *APOE* ε*4* genotype was associated with microstructural abnormalities of the white matter in late middle-aged adults (44). Studies using MRI have shown that *APOE* ε*4* is associated with an increase in WMH volumes (45, 46). In developing cerebral SVD, the expression of *APOE* ε*4*, but not of *APOE* ε*2 or of APOE* ε*3*, leads to BBB breakdown through the activation of an NF-kB/matrix metalloproteinase 9 pathway in pericytes (47). This causes cerebral SVD, allowing neurotoxic proteins from the blood to accumulate in the neuron. However, it is not clear whether *APOE* directly affects AD pathology or indirectly affects AD pathology through cerebral SVD.

Another genetic marker has been reported to be related to both the risk of AD and cerebral SVD. A study using a Dutch family-based cohort reported that the presence of APOE ε4, as well as *SORL1*, was associated with cerebral SVD and AD (48). The *SORL1* gene regulates APP processing, and SORL1 deficiency leads to increased levels of Aβ and enhances amyloid pathology in the brain (49). A recent meta-analysis study using the genomic-relatedness-matrix restricted maximum likelihood method found evidence of a shared genetic contribution between AD and cerebral SVD (50). They reported that one particular region on chromosome 17, that encompassed three genes (*ICT1/KCTD2/ATP5H*) was associated with both diseases. A pathway analysis identified four associated pathways involving cholesterol transport [gene ontology (GO)/phospholipid efflux, GO/cholesterol efflux, and GO/reverse cholesterol transport] and immune response (GO/negative regulation of nuclear factor kappa B transcription factor activity). Also, two polymorphisms (rs1801133 and rs1801131) in the methylenetetrahydrofolate reductase gene have been reported to correlate with elevated levels of plasma homocysteine as well as being associated with AD and vascular contributions to cognitive impairment (51). A recent study comparing 96 Caucasian cerebral SVD patients with 368 healthy controls reported a burden of truncation mutations in APP-Aß degradation genes (*EPHA1* p.M900V and p.V160A and *CD33* p.A14V). These genes were related with cerebral Aß accumulation, which has a protective effect on cerebral SVD (52).

## Relationship Between AD Biomarkers and Cerebral SVD

As cognitive function can be substantially affected by several factors, including AD pathology, contribution to cognitive impairment by cerebral SVD could be under- or overestimated depending on the cognitive reserve of each individual (39). For this reason, there is no linear correlation between AD pathology and cognitive impairment (53). It seems that cerebral SVD independently induces cognitive impairment with concurrent, synergistic exacerbation by AD pathology, resulting in MCI to dementia (25). Thus, the utilization of biological AD markers in place of its syndromal definition would be beneficial for evaluating the effect of cerebral SVD on AD development. The biomarkers that can be detected and quantified in AD are cerebral Aβ plaques, pathologic tau, and neurodegeneration (54). The biomarkers of cerebral Aβ plaques are low CSF Aβ42 and cortical amyloid positron emission tomography (PET) ligand binding (55). Biomarkers of pathologic tau are elevated CSF phosphorylated tau (p-tau) and cortical tau PET ligand binding (56). Biomarkers of neurodegeneration are cerebral hypometabolism on ^18^F-fluorodeoxyglucose (FDG) PET, and atrophy on MRI (57). The results of studies regarding relationships between AD biomarkers, including cerebral Aβ plaques, pathologic tau, and neurodegeneration, and cerebral SVD, are summarized in Table 1.

**Table 1:** Relationship between AD biomarkers and cerebral SVD.

**Participants (clinical diagnosis, *n*)**	**Study design**	**Type of SVD**	**AD biomarkers**	**Relationship**	**References**
50 subjects (CU = 50)	Cross-sectional	WMHs	Neurofibrillary tangle^a^	Positive^b^	(6)
83 subjects (AD = 34, MCI = 30, CU = 19)	Cross-sectional	WMHs	Cortical Aβ^c^	Positive	(8)
83 subjects (AD = 34, MCI = 30, CU = 19)	Cross-sectional	WMHs	Cortical metabolism^d^	Negative^e^	(9)
197 subjects (MCI = 159, CU = 38)	Cross-sectional	WMHs	CSF p-tau	Negative	(25)
184 subjects (unknown)	Cross-sectional	WMHs	Cortical Aβ	Positive	(53)
914 subjects (AD = 547; SCI = 337; VD = 30)	Cross-sectional	WMHs	CSF Aβ42	Negative	(58)
826 subjects (AD = 110, MCI = 195, SCI = 165, CU = 267, PD = 89)	Cross-sectional	WMHs	CSF Aβ40/CSF Aβ42/Cortical Aβ	Negative/Negative/Positive	(59)
		Lacunes	CSF Aβ40/CSF Aβ42/Cortical Aβ	No^f^/No/No	
56 subjects (CU = 56)	Cross-sectional	WMHs	CSF Aβ42	Positive	(60)
88 subjects (AD = 88)	Cross-sectional	Microbleeds	CSF Aβ40/CSF Aβ42	Negative/Negative	(61)
96 subjects (AD = 36, MCI = 18, CAA = 42)	Cross-sectional	WMHs	plasma Aβ40/plasma Aβ42	Positive/No	(62)
44 subjects (AD = 13, MCI = 17, CU = 14)	Cross-sectional	WMHs	Cortical Aβ	Positive	(63)
subjects (AD = 51, MCI = 18, SCI = 1, CU = 12)	Cross-sectional	WMHs	Neurofibrillary tangle	Positive	(64)
101 subjects (PPA = 82, CU = 19)	Cross-sectional	Microbleeds	CSF p-tau/Aβ42 ratio	Positive	(65)
200 subjects (SCI = 200)	Cross-sectional	Microbleeds	Cortical Aβ	Positive	(66)
282 subjects (CU = 282)	Cross-sectional	WMHs	Cortical Aβ	Positive	(67)
517 subjects (AD = 184, MCI = 118, SCI = 121, others = 94)	Cross-sectional	WMHs	CSF Aβ42/CSF p-tau	Positive/No	(68)
62 subjects (MCI = 36, CU = 26)	Cross-sectional	WMHs	CSF Aβ42	Positive	(69)
159 subjects (CU = 159)	Longitudinal	WMHs	Cortical Aβ	Positive	(70)
36 subjects (AD = 23, CU = 13)	Cross-sectional	WMHs	cortical p-tau	Positive	(71)
70 subjects (CU = 70)	Cross-sectional	WMHs	CSF Aβ42/CSF p-tau	Negative/Positive	(72)
424 subjects (MCI = 33, CU = 391)	Cross-sectional	WMHs	Cortical Aβ/Cortical tau	Positive/Negative	(73)
2367 subjects (unknown)	Cross-sectional	WMHs	Cortical atrophy	Positive	(74)
86 subjects (AD = 58, CU = 28)	Cross-sectional	WMHs	Cortical atrophy	Positive	(75)
72 subjects (CU = 72)	Cross-sectional	WMHs	Cortical Aβ/Cortical metabolism	Positive/Negative	(76)
60 subjects (AD = 21, MCI = 23, CU = 16)	Cross-sectional	WMHs	Cortical metabolism	Negative	(77)
819 subjects (AD = 193, MCI = 397, NC = 229)	Longitudinal	WMHs	CSF Aβ42	No	(78)
310 subjects (MCI = 310)	Cross-sectional	WMHs	CSF Aβ42/CSF t-tau	No/No	(79)
334 subjects (MCI = 60, CU = 274)	Longitudinal	WMHs	CSF Aβ42/CSF p-tau	No/No	(80)

*AD, Alzheimer's disease; SVD, small vessel disease; CU, cognitive unimpaired; WMHs, white matter hyperintensities; MCI, mild cognitive impairment; SCI, subjective cognitive impairment; VD, vascular dementia; PD, Parkinson's disease; PPA, primary progressive aphasia; CSF, cerebrospinal fluid*.

a Neurofibrillary tangle at autopsy;

b Positive relationship between Cerebral SVD and AD biomarker.;

c Cortical Aβ burden on amyloid PET image;

d Cortical glucose metabolism on ^18^F-FDG PET image;

e Negative relationship between Cerebral SVD and AD biomarker;

f *No significant relationship between Cerebral SVD and AD biomarker*.

Cerebral Aβ plaques and pathologic tau indicate specific neuropathologic changes that define AD, whereas neurodegeneration is not specific to AD (54). CSF and plasma Aβ levels or Aβ PET imaging with ^11^C-PIB, ^18^F-florbetapir, ^18^F-florbetaben, or ^18^F-flutemetamol have been used for measuring cerebral Aβ pathology. Several cross-sectional or longitudinal studies have shown an association between WMHs and Aβ plaques. A study using the memory clinic-based Amsterdam Dementia Cohort reported associations of WMHs and CBMs with CSF Aβ42 (58). Two other CSF analysis studies showed that a higher WMH burden correlated with lower levels of Aβ in the CSF (59, 60). A study with CSF markers in AD patients showed that patients with cortical microbleeds had lower levels of CSF Aβ40 and Aβ42 than those without microbleeds after adjusting age, sex, APOE ε4 presence, and WMH burden (61). A study using specific enzyme-linked immunosorbent assays reported that WMHs were significantly associated with plasma Aβ40 and Aβ42 levels in an AD and MCI population (62). A study with immunohistochemistry also showed a positive correlation between the cerebral Aβ burden at autopsy and the WMH volume score in T2 MRI in a cohort of older adults (6). Furthermore, a study with Aβ PET imaging using information extracted from the ADNI database showed that WMHs were more highly correlated with cerebral Aβ burden than any of the standard AD imaging biomarkers (53). A study using Aβ PET and functional MRI revealed that whole-brain WMHs and cerebral Aβ deposition were significantly higher in AD patients than in controls, showing that increased WMH burden disrupts the functional connectivity of the prefrontal and temporal cortices (63). A Clinicopathological study in the United States with 82 participants from the National Alzheimer's Coordinating Center's Data Sets found a direct association between total volume of WMH and increased risk of exhibiting AD neuropathology (defined as frequent neuritic plaques and Braak stage III-VI at autopsy) (64). In agreement with these previous studies, our study group also found that the high-WMHs group exhibited a greater cerebral Aβ burden compared with the low-WMHs group and that the cerebral Aβ burden was positively correlated with WMH burden (8). In addition, one multicenter cohort study with CSF analysis revealed that CMBs were more frequent in patients with AD pathology than without AD pathology (65) and another study with Aβ PET showed that parietal CMBs were associated with cerebral Aβ burden (66). Although these findings suggest that cerebral SVD may play a significant role in AD development, there is still a possibility that cerebral Aβ deposition will cause white matter alteration (67–69), due to the limitations of the cross-sectional study design. A recent longitudinal study with 159 cognitively normal participants from the ADNI data set showed that an increased baseline burden was associated with faster cerebral Aβ accumulation in 2-years follow-up period, suggesting WMH contributes to the development of AD (70).

Several studies have evaluated the relationship of cerebral SVD with tau pathology, using CSF tau, immunohistochemistry of phosphorylated tau, and tau PET imaging with ^18^F-AV 1451, ^18^F-FDDNP, or ^18^F-THK-523. The results of studies on the association between cerebral SVD and tau pathology are conflicting. An immunohistochemistry study reported that cortical tau load at autopsy was associated with WMH burden in 36 cerebral hemispheres (71). A recent study using diffusion tensor imaging revealed a decrease in fractional anisotropy, which is an index of the WMH burden, significantly correlated with AD biomarkers, including CSF p-tau (72). Furthermore, a longitudinal study of 197 patients for 5.7 years showed that MCI patients with both pathological levels of phosphorylated tau and WMHs at baseline progressed more rapidly toward AD. This suggested that cerebral SVD and tau pathology likely have independent but synergistic effects on the reduction of the cognitive reserve capacity of the brain (25). However, another recent studies with tau PET imaging revealed that WMHs were not significantly associated with increased p-tau burden (73).

Neurodegeneration can result from many causes and is not specific to AD. However, the combination of an MRI or ^18^F-FDG PET study with AD biomarkers provides a much more robust prediction of future cognitive decline than an abnormal amyloid study alone (54). A large population-based study found that WMHs contributed to brain atrophy patterns in regions associated with AD (74). A study using MRI revealed an interaction between medial temporal lobe atrophy and WMHs, suggesting that cerebral SVD and AD pathology act in synergy in AD (75). While cerebral atrophy on MRI likely reflects cumulative loss and shrinkage of the neuropil, ^18^F-FDG PET probably indicates both cumulative losses of the neuropil and functional impairment of the neurons (81). Typical findings of ^18^F-FDG PET in AD patients are decreased glucose metabolism in temporal and parietal cortices, posterior cingulate, and precuneus (82), whereas more advanced AD results in decreased glucose metabolism up to the frontal cortex (83). Our study group reported that WMH burden was negatively correlated with regional glucose metabolism in the bilateral frontal, temporal, and parietal cortices, and limbic lobes in patients with cognitive impairment (9). The decreased cerebral glucose metabolism by WMHs is known to be due to disruption of functional connectivity. A study using the connectivity change score on MRI also revealed that in cognitively unimpaired subjects, those with more impaired connectivity of their gray matter due to WMHs also had lower glucose metabolism (76). Another cohort study revealed that disruption of limbic white matter pathways caused decreased glucose metabolism in the parietal and temporal cortices and posterior cingulate in patients with cognitive impairments (77). These findings suggest that cerebral SVD has a similar pattern of AD with decreased cerebral glucose metabolism and may be a cause of cognitive impairment in AD.

Although most of the studies evaluating the relationship have shown a significant relationship between cerebral SVD and AD biomarkers, including cerebral Aβ, pathologic tau, and neurodegeneration, the results of some studies have not (78–80). The conflicting results regarding the relationship between cerebral SVD and AD biomarkers can be explained by studies not adjusting the gray matter volume or by the use of different methods to measure cerebral SVD. Additionally, various definitions of cerebral SVD may have led to conflicting results (11).

## Mechanisms Linking Cerebral SVD to AD

Although the underlying mechanism of cerebral SVD to induce AD pathology is still unclear, it can be explained by chronic cerebral hypoperfusion (CCH) or BBB disruption from the cerebral SVD (84). Firstly, cerebral SVD restricts the vessel lumen, causing CCH in white matter where collateral vessels do not develop, resulting in ischemic damage. This leads to repetitive and selective apoptosis of oligodendrocytes that are vulnerable to ischemia and eventually to degeneration of myelinated fibers (85). Thus, CCH causes neurodegeneration of white matter through neuronal energy failure, which is further facilitated by proinflammatory cytokines via the production of reactive oxygen species and activated microglial cells (86, 87). Additionally, CCH can accelerate cerebral Aβ deposition (88). Our study group showed that CCH could aggravate the AD pathology, including cerebral Aβ and p-tau, and selectively decrease the neuronal activity of the limbic system in rats (89). Another study with mice overexpressing a mutant form of the human APP revealed that CCH by bilateral common carotid artery surgery increased cerebral Aβ accumulation and promoted cognitive impairment in combination with APP gene mutations (90). It seems that CCH increases Aβ deposition by up-regulating APP processing because overexpression of the β-secretase gene on the 2nd day and overexpression of the APP gene on the 7th and 30th was found after global cerebral ischemia in a longitudinal study with rats (91). Furthermore, CCH increases hypoxia-induced factor-1 expression, which not only activates the promoter of β-secretase but also increases the expression of β-secretase (92). In a vicious circle, cerebral SVD promotes Aβ accumulation, thereby promoting the restriction of small vessel lumen, resulting in irreversible neuronal damage (61). It has also been reported that CCH causes cognitive dysfunction by reducing protein O-GlcNAcylation and promoting tau phosphorylation in the mouse model (93).

Secondly, another possible mechanism to induce AD pathology by cerebral SVD is BBB disruption. Several studies using MRI and postmortem brain pathology have reported the presence of BBB dysfunction in AD patients, suggesting that BBB disruption could affect AD development independent of cerebral Aβ pathology (94, 95). As cerebral Aβ is primarily cleared by a vascular path in BBB, the disruption in neurovascular integrity is thought to contribute to inducing AD pathology, resulting in the onset and progression of cognitive decline (96). A recent study using a CSF biomarker of BBB-associated capillary mural cell pericytes, and which examined the regional BBB permeability using dynamic MRI, showed that patients with AD have BBB disruption combined with cerebral SVD in the hippocampus, regardless of cerebral Aβ plaque and pathologic tau, suggesting that BBB disruption is an early biomarker for AD (97). Following BBB disruption, neurotoxic Aβ peptides are released from the circulatory system, which eventually exacerbates ischemic neurons leading to neuronal death (98). In addition, the cerebral Aβ plaque narrows the small vessel lumen, worsening ischemia, and causing secondary neuronal death (99). This vicious cycle caused by this BBB disruption may cause a loss of the neuronal network connectivity in combination with CCH from cerebral SVD and may advance cognitive impairment in AD.

## Future Prospective

Many epidemiological, genetic, and clinical-pathological studies support the association of cerebral SVD in developing AD. However, the molecular mechanisms linking cerebral SVD to AD pathogenesis are not fully understood. Some investigators have hypothesized that the primary cause for developing AD is cerebral SVD (8, 97, 98). Several clinical and animal studies support this ischemic hypothesis on AD development in terms of cerebral SVD, causing CCH. Normal aging decreases the cerebral perfusion by about 20% when comparing 60-year-olds to those that are 20 years old (100). In addition to decreased cerebral perfusion in normal aging, additional decrease in cerebral perfusion is more likely to damage neurons that are vulnerable to ischemia (101). Studies using animal models have reported that hippocampus is particularly vulnerable to ischemia (102). In particular, CA1 was shown to be highly damaged after ischemia, while CA3 and granule cells were conserved in studies using rodent models (103). The selective injury of the hippocampus by CCH would cause the disconnection of the hippocampal-cortical network, thereby reducing the neuronal activity of the temporal and parietal lobes, which in turn causes secondary neuronal degeneration (104, 105). A study using a flow-enhanced signal intensity technique of MRI showed that decreased perfusion of the hippocampus was related with loss of spatial memory, suggesting that CCH of the hippocampus is associated with cognitive impairment in older individuals (100). After the hippocampus is selectively damaged, the cerebral cortices, which are functionally closely connected with the hippocampus, are affected, and in turn the prion-like tau spreading is facilitated by neural activity (106). In addition, cerebral SVD itself, which occurs in white matter tracts, can contribute to cognitive impairment, with several PET studies with ^18^F-FDG indicating that secondary neuronal degeneration with disconnection is a major factor in early posterior hypometabolism in AD (105, 107). The cingulum bundle, a prominent tract in white matter, is disrupted by cerebral SVD, resulting in decreased glucose metabolism in a large connected network, including the whole memory circuit of Papez and the posterior association cortex (104). A recent study with 503 subjects revealed that the interaction between cerebral SVD and hippocampal volumes explained the memory decline, suggesting memory impairment is a heterogeneous condition with different pathologies (108).

However, there are some limitations to the ischemic hypothesis on AD development. There is a lack of direct evidence regarding the mechanisms that explain the development of AD pathology by cerebral SVD. It is also unclear whether cerebral SVD generates AD pathology directly or in combination with other causes. In contrast, AD pathology may affect vascular and endothelial function, which may contribute to the development of cerebral SVD and, potentially, to failure of eliminating abnormal neurotoxic proteins, such as Aβ and phosphorylated tau, from the brain (109). Furthermore, the cause of cerebral SVD has not yet been clarified. In addition to hypertension and hypercholesterolemia, systemic diseases, such as disturbances of the brain-gut-microbiota axis and chronic inflammation, which have recently been reported as causes of AD, may contribute to or worsen cerebral SVD development, and in turn affect AD development (110, 111). Nevertheless, as described in this review, recent studies on the relationship between cerebral SVD and AD development further support the hypothesis that cerebral SVD contributes to AD development. The mechanism by which cerebral SVD affects AD development, along with other complex causes, and how to prevent AD development or slow AD progression by inhibiting this process should be studied in the future.

## Conclusion

In summary, there is substantial epidemiologic, genetic, and clinical evidence regarding the association between cerebral SVD and AD. Cerebral SVD may contribute to cognitive impairment through cerebral Aβ accumulation and play a significant role in AD development. Further investigation is required to understand the mechanistic pathways for the contribution of cerebral SVD on the development of AD pathology. Further longitudinal studies regarding cerebral SVD progression should result in new insights regarding the etiology and treatment of AD.

## Author Contributions

HK and JH wrote manuscript and carried out the subsequent revisions. JJ searched literatures and prepared the supporting material. All authors contributed to the article and approved the submitted version.

## Conflict of Interest

The authors declare that the research was conducted in the absence of any commercial or financial relationships that could be construed as a potential conflict of interest.
